# Marine-Derived Phosphoeleganin and Its Semisynthetic Derivative Decrease IL6 Levels and Improve Insulin Signaling in Human Hepatocellular Carcinoma Cells

**DOI:** 10.3390/ijms25116039

**Published:** 2024-05-30

**Authors:** Ayewa L. Agognon, Marcello Casertano, Alessio Vito, Sonia Orso, Serena Cabaro, Federica Mormone, Cristina Morelli, Giuseppe Perruolo, Pietro Formisano, Marialuisa Menna, Concetta Imperatore, Francesco Oriente

**Affiliations:** 1 Department of Translational Medicine, Federico II University of Naples and URT “Genomic of Diabetes” of Institute of Experimental Endocrinology and Oncology, National Council of Research (CNR), Via Pansini 5, 80131 Naples, Italy; ayewa.agognon@gmail.com (A.L.A.); sonia.orso@hotmail.com (S.O.); serena.cabaro@unina.it (S.C.); federica.mormone@outlook.it (F.M.); cristinamorelli@tim.it (C.M.); giuseppe.perruolo@unina.it (G.P.); fpietro@unina.it (P.F.); 2 Department of Pharmacy, University of Naples “Federico II”, Via D. Montesano 49, 80131 Napoli, Italy; marcello.casertano@unina.it (M.C.); alessio.vito@unina.it (A.V.); cimperat@unina.it (C.I.); 3 Center for Basic and Clinical Immunology Research (CISI), University of Naples Federico II, 80131 Naples, Italy

**Keywords:** phosphoeleganin, marine natural products, HepG2 cells, insulin signaling, cytokines

## Abstract

Marine natural products constitute a great source of potential new antidiabetic drugs. The aim of this study was to evaluate the role of phosphoeleganin (PE), a polyketide purified from the Mediterranean ascidian *Sidnyum elegans*, and its derivatives PE/2 and PE/3 on insulin sensitivity in human hepatocellular carcinoma (HepG2) cells. In our experiments, insulin stimulates the phosphorylation of its receptor (INSR) and AKT by 1.5- and 3.5-fold, respectively, whereas in the presence of PE, PE/2, and PE/3, the insulin induced INSR phosphorylation is increased by 2.1-, 2-, and 1.5-fold and AKT phosphorylation by 7.1-, 6.0-, and 5.1-fold, respectively. Interestingly, PE and PE/2 have an additive effect on insulin-mediated reduction of phosphoenolpyruvate carboxykinase (PEPCK) expression. Finally, PE and PE/2, but not PE/3, decrease interleukin 6 (IL6) secretion and expression before and after palmitic acid incubation, while in the presence of high glucose (HG), only PE reduces IL6. Levels of other cytokines are not significantly affected by PE and its derivates. All these data suggest that PE and its synthetic-derived compound, PE/2, significantly decrease IL6 and improve hepatic insulin signaling. As IL6 impairs insulin action, it could be hypothesized that PE and PE/2, by inhibiting IL6, may improve the hepatic insulin pathway.

## 1. Introduction

Insulin resistance (IR) is characterized by hyperglycemia due to the failure of insulin-dependent cells, such as hepatocytes, myocytes, and adipocytes, to properly respond to normal concentrations of circulating insulin, the major glucose-lowering hormone. In the liver, once secreted by pancreatic β-cells, insulin binds to its receptor (INSR) and induces complex signaling cascades through its substrates [1,2,3,4]. This leads to the activation of two main pathways: one corresponds to the activation of phosphatidylinositol 3-kinase (PI3K) and the Protein Kinase B (AKT) pathway, which promotes metabolic activities such as glycogen synthesis and gluconeogenesis inhibition [1,2]. The other, called the extracellular signal-regulated kinase (ERK) 1/2 pathway, corresponds to the activation of cell proliferation and differentiation. 

To date, mechanisms that contribute to hepatic IR are still not fully understood. Among the several molecules that can impair insulin signal transduction in hepatocytes, cytokines have been shown to increase in diabetic patients and induce insulin resistance. In particular, IL6, CCL2 (also called MCP1), and IL1β inhibit insulin signaling in HepG2 cells, and this effect is, in part, prevented by the antidiabetic drug metformin, which, however, can cause side effects [5,6,7,8,9,10]. Thus, the study of the molecular mechanisms underlying the pathogenesis of hepatic IR is a major challenge in modern biomedical research that aims to increase knowledge on several metabolic disorders, such as non-alcoholic fatty liver disease (NAFLD) and dysfunction-associated fatty liver disease (MAFLD). Similarly, the discovery of new chemical entities that can modulate insulin sensitivity in liver cells is critical for the development of new drugs for the prevention and treatment of liver disease progression. In this context, capturing and exploiting the biological activity of natural products (NPs) offers particularly favorable features, such as their huge scaffold diversity and structure complexity. In addition, a NP-informed strategy addresses the high demand in target-based early drug discovery for three-dimensional structures rich in *sp^3^* hybridized carbons, with a high number of stereogenic centers and good solubility, which are favorable key properties in bioactive compound discovery. Particularly, in recent years, interest in the less-explored marine world has significantly grown thanks to technological advances that have made it possible to investigate the biodiversity of this environment. Hence, marine NPs have arisen as a new and sustainable resource for drug leads with which novel mechanisms of action are associated [11,12,13,14]. Marine invertebrates in particular offer an excellent opportunity to study diverse and unique compounds not easily accessible from other sources and potentially useful for the treatment of chronic diseases and glycemic control [15]; marine invertebrates are the source of most approved commercial marine-based drugs [11,16]. 

**Figure 1:**
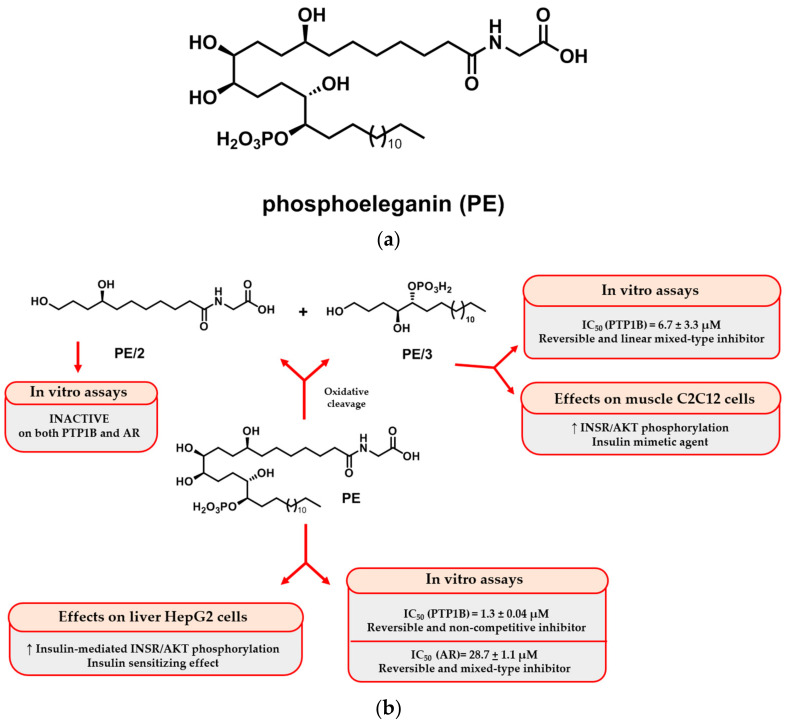
(**a**) The marine polyketide phosphoeleganin (PE), purified from the Mediterranean ascidian *S. elegans.* (**b**) Previously reported pharmacological effects of PE and its semisynthetic derivatives PE/2 and PE/3. For the oxidative cleavage, reactions and conditions are (i) NaIO_4_, MeOH, rt, 3 h; (ii) NaBH_4_, 0 °C, 1 h.

We recently purified a novel phosphorylated polyketide, phosphoeleganin (PE) (Figure 1a), from the Mediterranean ascidian *Sidnyum elegans* [17,18]. PE was shown to be a dual inhibitor of Protein Tyrosine Phosphatase 1B (PTP1B) and Aldose Reductase (AR) enzymes [19]. Both enzymes are involved in the onset and progression of chronic multifactorial diseases, such as type 2 diabetes mellitus (T2DM), and, thus, they are now emerging targets for antidiabetic leads [20,21]. The very significant inhibiting effect of PE on PTP1B activity also causes an increase in INSR and AKT phosphorylation levels in human hepatocellular carcinoma (HepG2) cells. Furthermore, the phosphorylation levels of INSR and AKT increased in insulin-stimulated HepG2 cells but not in non-stimulated HepG2 cells, revealing that PE is an insulin-sensitizing agent. Thus, this molecule represents a potential lead for the design of multitarget drugs that can counteract IR and the onset of diabetic complications [19].

In a subsequent study, aiming to investigate the structural requirements underlying the inhibition of PTP1B and AR enzymes, the structure of the natural metabolite PE was chemically manipulated to give small-size and less complex fragments in order to better explore the biologically relevant chemical space of PE. The oxidative cleavage of PE at the C11-C12 diol system was performed, affording the derivatives PE/2 and PE/3 (Figure 1b), which were both tested against PTP1B and AR. Unfortunately, all the fragments lost their activity on AR; on the other hand, the phosphorylated fragment PE/3, likewise PE but unlike PE/2, retained a potent PTP1B inhibiting activity with a reversible and mixed-type noncompetitive mechanism. It was also shown that in C2C12 cells treated with PE/3 alone, the phosphorylation levels of both INSR and AKT kinase increased compared with control cells, suggesting that PE/3 acts as an insulin-mimetic agent when tested on muscle cells [22].

Hence, based on the above, the aim of this study was to explore the influence of the natural molecule PE and its two semisynthetic fragments PE/2 and PE/3 on the insulin sensitivity of hepatocellular carcinoma cell lines (HepG2). 

Due to the limited availability of human primary hepatocytes, HepG2 cells have extensively been used to study hepatic insulin signaling, as they appear to be closer to the in vivo situation despite their tumorigenic origin and show specific hepatocyte morphological characteristics. Sefried et al. have shown an increase in insulin-mediated Akt phosphorylation [23] in HepG2 cells, and these findings are consistent with a reduction of *PEPCK* expression observed by Won-Mo Yang [24]. In parallel, several polyphenols, such as resveratrol and morin, improve glucose metabolism in these cells [25,26]. Consequently, the HepG2 cell line could provide a valuable tool for identifying drug candidates that target the insulin pathway in the liver [27].

## 2. Results

### 2.1. Isolation and Purification of PE and Semisynthetic Approach for Obtaining PE/2 and PE/3

A sample of PE was purified using our previously reported protocol based on an exhaustive extraction of samples of *S. elegans* collected in the Neapolitan Bay of Pozzuoli and the application of sequential chromatographic separations and purifications of the obtained extract [17]. This protocol afforded PE in a pure form. An aliquot of the purified polyketide was cleaved at the 1,2-anti diol system C11-C12 by treatment with sodium periodate (NaIO_4_) and, successively, with the reducing agent sodium borohydride (NaBH_4_). After purification by RP-HPLC, the semisynthetic derivatives PE/2 and PE/3 were also obtained in pure form. The purity of all compounds was assured by NMR analysis; their identity was unequivocally established by comparing their spectrometric and spectroscopic properties with the previously reported data [17,22]. 

### 2.2. Role of Phosphoeleganin and Its Derivatives in Insulin Signaling

**Figure 2:**
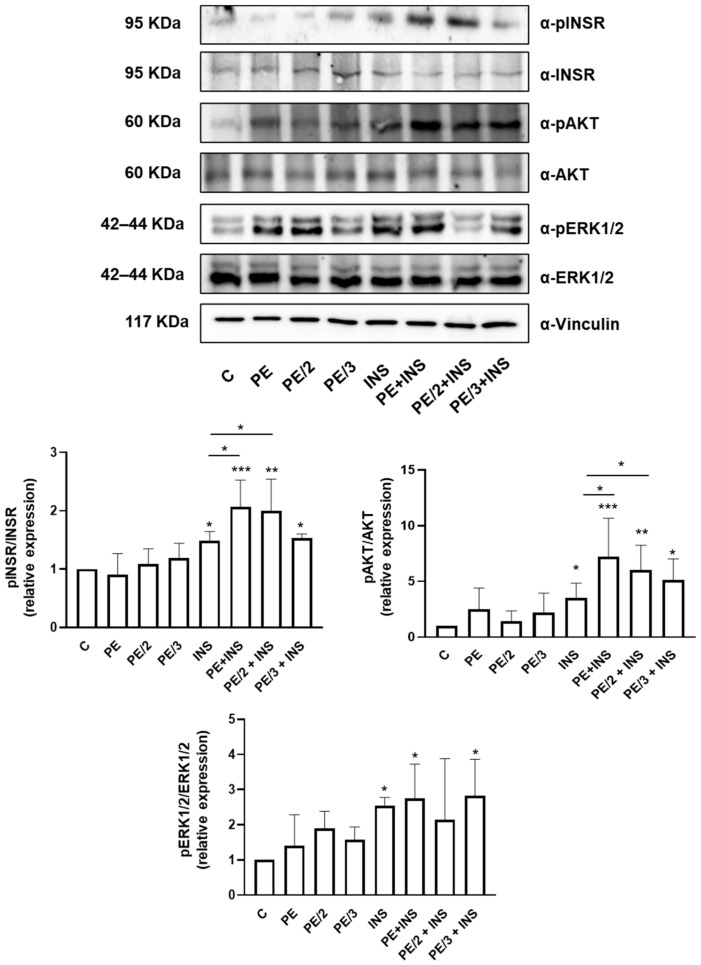
Effect of PE, PE/2, and PE/3 on insulin receptor (INSR), AKT, and ERK1/2 phosphorylation. HepG2 cells were incubated with PE, PE/2, and PE/3 (25 µM) for 4 h and then stimulated with insulin (100 nM) for 10 min. The protein expression of pINSR, pAKT, and pERK1/2 was analyzed by Western blotting. INSR, AKT, and ERK1/2 bands were taken from a parallel gel loaded with the same lysates. Vinculin antibody was used for normalization. The autoradiographs shown are representative of three different experiments and subjected to densitometric analysis. Bars represent the mean ± SD of three independent experiments. Asterisks denote statistical differences (* *p* < 0.05; ** *p* < 0.01; *** *p* < 0.001).

To investigate the role of PE and its derivatives (PE/2 and PE/3) on hepatic insulin signaling, HepG2 cells were treated with PE, PE/2, and PE/3 in the presence or absence of insulin. As shown in Figure 2, INSR tyrosine phosphorylation (pINSR) did not significantly change in the presence of PE and its derivatives. However, while insulin stimulated pINSR by 1.5-fold compared to the control cells, PE, PE/2, and PE/3 ameliorated the effect of insulin increasing INSR tyrosine phosphorylation by 2.1-, 2-, and 1.5-fold, respectively. Interestingly, the additive effect of PE and PE/2 on insulin on pINSR was statistically significant compared to treatment with insulin alone. Conversely, PE/3 did not modify the amount of phosphorylated INSR. 

Similarly, treatment of HepG2 cells with PE, PE/2, and PE/3 did not significantly affect the amount of phosphorylated AKT. In contrast, insulin increased pAKT by 3.5-fold compared to the untreated cells, but in the presence of PE, PE/2, and PE/3, we observed a 7.1-, 6.0-, and 5.1-fold increase in AKT phosphorylation, respectively. In this case, the improvement mediated by PE and PE/2 on insulin-stimulated AKT phosphorylation was statistically significant compared to treatment with insulin alone. 

Surprisingly, the amount of phosphorylated ERK1/2 significantly increased in the presence of insulin alone or with PE and PE/3, compared to the control cells. However, no difference was observed between PE + insulin and PE/3 + insulin with respect to the insulin-stimulated HepG2. Total levels of INSR, AKT, and ERK1/2 did not change in the presence of insulin, PE, PE/2, or PE/3. 

**Figure 3:**
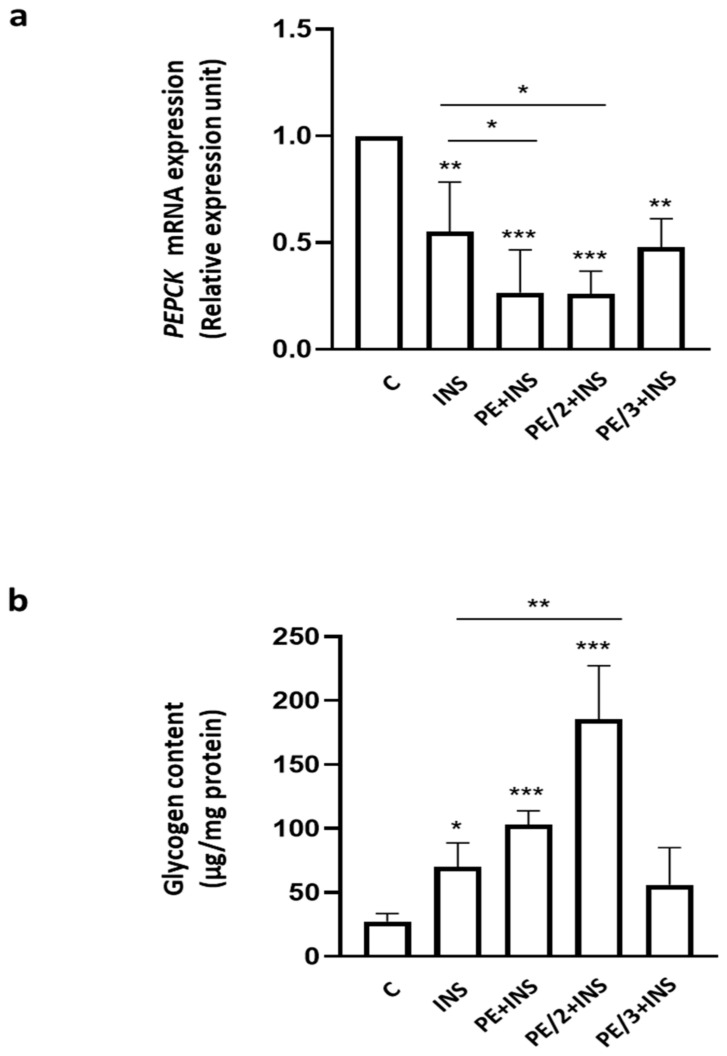
Effect of PE, PE/2, and PE/3 on PEPCK expression and glycogen content. (**a**) HepG2 cells were incubated with PE, PE/2, and PE/3 (25 µM) and insulin (100 nM) for 8 h. *PEPCK* mRNA levels were evaluated by real-time RT-PCR analysis. Data were normalized by the amount of *beta-ACTIN*, used as an internal control. Bars represent the mean ± SD of three independent experiments, each performed in triplicate. (**b**) HepG2 cells were incubated with PE, PE/2, and PE/3 (25 µM) and insulin (100 nM) for 3 h. Glycogen content was measured as described in the Section 4. Asterisks denote statistical differences (* *p* < 0.05; ** *p* < 0.01; *** *p* < 0.001).

To further evaluate the hepatic insulin downstream effects, we measured the expression of phosphoenolpyruvate carboxykinase (PEPCK), one of the main gluconeogenic enzymes, and evaluated glycogen content. As shown in Figure 3a, insulin decreased *PEPCK* mRNA by 45%, and PE and PE/2 improved the insulin effect, reducing the levels of this enzyme by 73 and 74%, respectively. In parallel, insulin alone stimulated glycogen synthesis by 2.6-fold, while this synthesis rose to 6.9-fold in the presence of PE/2 (Figure 3b). A tendency for PE to ameliorate the insulin effect on glycogen content was observed.

### 2.3. Role of Phosphoeleganin and Its Derivatives on Cytokine Secretion and Expression

**Figure 4:**
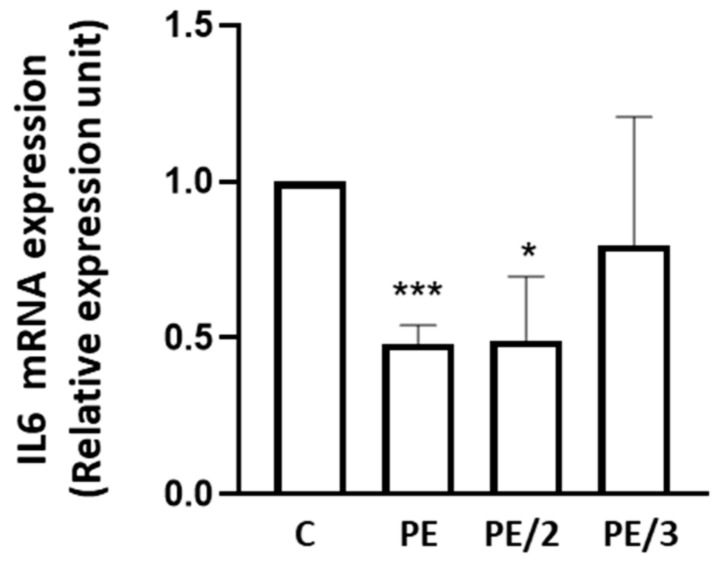
Effect of PE, PE/2, and PE/3 on IL6 expression. HepG2 cells were incubated with PE, PE/2, and PE/3 (25 µM) for 4 h. *IL6* mRNA levels were evaluated by real-time RT-PCR analysis. Data were normalized by the amount of *beta-ACTIN*, used as an internal control. Bars represent the mean ± SD of three independent experiments, each performed in triplicate. Asterisks denote statistical differences (* *p* < 0.05; *** *p* < 0.001).

As several cytokines can negatively modulate insulin signaling [28,29,30,31,32], we measured the basal cytokine secretion profile in culture medium obtained from HepG2 cells in the presence of PE and its derivatives (Table 1). We observed a significant reduction (~26%) only in IL6 secretion after incubation with PE and PE/2; the reduction of IL8 secretion was also observed after incubation with PE/2. The effect on IL6 secretion also agrees with the observed decrease in IL6 expression after incubation with PE and PE/2 and not with PE/3 (Figure 4). Thus, we particularly focused our attention on IL6.

**Figure 5:**
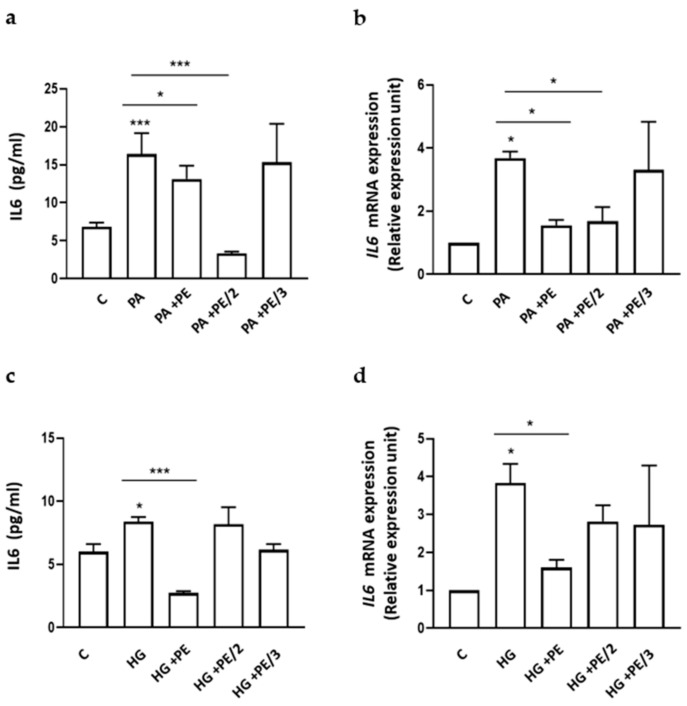
Effect of PE, PE/2, and PE/3 on IL6 secretion and expression in the presence of palmitic acid (PA) (**a**,**b**) or high glucose (HG) (**c**,**d**). HepG2 cells were incubated with PA (0.5 mM) for 8 h or with HG (60 mM) for 24 h with or without PE, PE/2, and PE/3 (25 µM). IL6 secretion was determined in the conditioned media by using a custom Human Magnetic Luminex Assay. *IL6* mRNA levels were evaluated by real-time RT-PCR analysis. Data were normalized by the amount of *beta-ACTIN*, used as an internal control. Bars represent the mean ± SD of three independent experiments, each performed in triplicate. Asterisks denote statistical differences (* *p* < 0.05; *** *p* < 0.001).

Therefore, we evaluated the IL6 secretion and expression levels after exposure of HepG2 cells to palmitic acid (PA) and high glucose (HG), which are known to cause metabolic derangement and participate in the pathogenesis of obesity, insulin resistance, and steatosis [32,33]. The PA- and HG-exposed HepG2 cells were then treated with PE, PE/2, or PE/3. As shown in Figure 5, PA increased IL6 secretion (a) and expression (b) by 2.4- and 3.7-fold, respectively, and this effect was reverted by PE and PE/2, but not PE/3. Interestingly, HepG2 cells treated with HG featured a 1.4- and 3.8-fold increase in IL6 secreted and mRNA levels, respectively. However, only PE reverted the HG effect (Figure 5c,d). 

## 3. Discussion

Insulin is a hormone secreted by the beta cells of the pancreas acting through a cell surface receptor (INSR) expressed in a variety of cell types, including hepatocytes, skeletal muscle, and adipocytes. The binding of insulin to its receptor induces the activation of the PI3K/AKT and ERK1/2 pathways. While PI3K/AKT activation mainly stimulates hepatic metabolic activities, such as glycogen synthesis, lipogenesis, and gluconeogenesis inhibition, ERK1/2 activation promotes cell proliferation and differentiation [34]. Evidence indicates that dysfunctions in the insulin signaling pathway cause IR, which is pivotal in the pathogenesis, development, and progression of NAFLD/MAFLD disease [35]. Although beneficial effects of antidiabetic drugs, such as thiazolidinediones or metformin, have been reported in IR and more generally in NAFLD/MAFLD treatment, these drugs may also present important side-effects such as weight gain and fluid retention, limiting their applications and potentially reducing patient compliance [36,37,38]. Thus, further basic science is necessary to provide new insights into molecular mechanisms involved in the improvement of insulin sensitivity in the liver, thus increasing understanding of these metabolic diseases’ pathogenesis. In parallel, the discovery of new tools for the treatment of NAFLD/MAFLD and related diseases might be of extreme importance. To achieve these aims, the availability of suitable chemical means is also a main concern. Indeed, a key tool in enabling the advancement of knowledge of biological processes and at the same time providing a basis for new drug discovery are small organic molecules, which can be used as “probe molecules” to both understand and treat diseases. In this view, marine NPs, in particular, are now emerging as novel lead structures for identifying and designing potential drugs against IR and its associated complications, such as T2D, obesity, metabolic syndrome, and cardiovascular complications [16,39,40]. As reported above, PE acts as an insulin-sensitizing agent on HepG2 cells moving within the cell’s cytoplasm, where it can inhibit PTP1B and enhance the insulin signaling pathway [19]. That PE can cross the plasma membrane is rather unexpected due to the presence in its structure of the charged phosphate group; however, the involvement of fatty acid transporters or endocytosis processes could be hypothesized, justifying our findings. This encouraged the exploration of the biologically relevant chemical space of this compound for a deeper pharmacological characterization through small-size and less complex derivatives, PE/2 and PE/3, obtained by oxidative cleavage of PE. When screened against PTP1B, only PE/3 was effective as a reversible and mixed-type noncompetitive inhibitor, demonstrating that the presence of a phosphate group is crucial for PTP1B inhibition. However, the IC_50_ of PE/3 was an order of magnitude lower than that of PE (IC_50_ = 6.7 ± 3.3 µM for PE/3 vs. IC_50_ = 1.3 ± 0.04 µM for PE), thus suggesting the importance of the whole structure for the enzyme inhibition. Moreover, PE/3 was demonstrated to act as an insulin mimetic agent when tested on murine myoblast C2C12 cells [22]. 

Based on these premises, in the current work, the effects of PE, PE/2, and PE/3 on the regulation of hepatic insulin signaling have been investigated in HepG2 cells. As for the INSR/AKT signaling, our results confirm our previous finding that highlights PE as an insulin-sensitizing agent in HepG2 cells. Interestingly, remarkable differences in the effects of the two fragments of PE have been observed. Indeed, only PE/2 was able to increase INSR/AKT phosphorylation in the presence of insulin, acting as an insulin-sensitizing agent such as PE, although it was not able to inhibit PTP1B. On the contrary, the phosphorylated fragment PE/3, previously evidenced as an insulin mimetic agent in muscle cells and able to significantly inhibit PTP1B [22], was inactive on insulin signaling in HepG2. None of the tested compounds affected the INSR/ERK1/2 pathway. 

To corroborate these results and as read out for metabolic insulin action in hepatic cells, we have evaluated the effects of PE and its derivatives on *PEPCK* expression, an enzyme that plays an important role in gluconeogenesis, and on glycogen production. 

PE and PE/2 significantly improve the insulin effect on *PEPCK* mRNA expression, while the additive effect of insulin on glycogen synthesis is significant only for PE/2, although we observed a tendency for PE to ameliorate the insulin effect on glycogen content.

The fact that the fragment, and not the original molecule PE, is active in this experiment can be explained by supposing that the whole molecule contains a region, not present on PE/2, which can negatively regulate its INSR/AKT downstream activity and control the activating effect. However, further studies are needed to clarify this hypothesis. 

An impairment of hepatic insulin action is caused by many factors. In particular, several lines of evidence support a role for cytokines and chemokines in the pathogenesis of insulin resistance [28,41,42]. Therefore, to estimate the effects of PE and its derivatives on cytokines and related proteins, we have first evaluated if these compounds could affect their basal secretion. Our results indicate that PE and PE/2, unlike PE/3, reduce IL6 secretion and gene expression. PE/2 is also able to significantly decrease IL8 secretion, whereas the secretion of the other cytokines and related proteins (IL1β, IL1ra, IL6R, CCL2, CCL3, Inhibin, and VEGF) is not affected by PE, PE/2, and PE/3. Thus, we have particularly focused our attention on IL6. 

Both in vitro cell culture and in vivo animal studies have indicated that glucolipotoxicity has been strictly associated with hepatic insulin resistance [5,29,32,33]. On this basis, we have exposed HepG2 cells to high concentrations of glucose (HG, glucotoxicity) and palmitic acid (PA, lipotoxicity), the most abundant saturated fatty acid. In these conditions, we observed that PE/2 counteracts PA-mediated IL6 secretion, whereas it has no effect in the presence of HG. We can therefore speculate that while the whole molecule is required to reduce IL6 levels in the case of glucotoxicity, the PE/2 fragment might be sufficient in the presence of lipotoxicity. 

These data are particularly interesting as they indicate a specific role of PE and PE/2 on IL6, which has been described to inhibit the INSR/AKT pathway and then induce insulin resistance in the mouse liver and in HepG2 cells [43]. 

**Figure 6:**
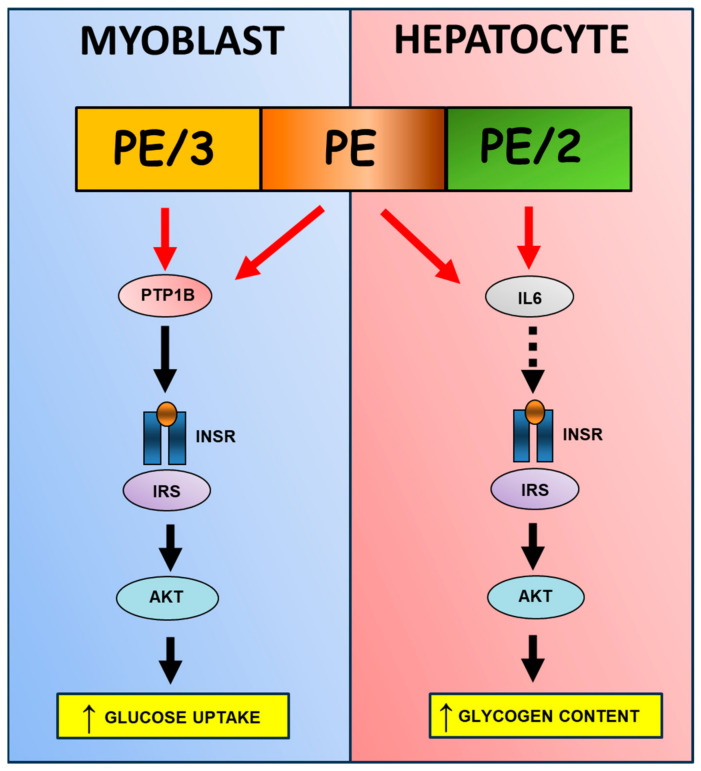
Scheme illustrating PE, PE/2, and PE/3 action in myoblast and hepatocyte. PE/3 exhibits a high inhibitory activity towards PTP1B, which enhances the INSR/AKT pathway and leads to increased glucose uptake in muscle cells. However, considering PE/2’s inability to act through PTP1B, we may suppose that its improvement of hepatic insulin resistance could be mediated by the inhibition of IL6 (black dashed arrow). Finally, PE possibly targets both IL6 and PTPB1, hence its amelioration of the insulin pathway in both muscle and hepatic cells (red arrows refer to negative regulation, black arrows refer to positive regulation).

Overall, our results indicate that PE/3, unlike PE/2, does not improve insulin signaling in HepG2 cells, despite being a potent inhibitor of PTP1B. Indeed, as we have previously shown, PE/3 acts as an insulin mimetic agent in muscle cells [22]. Conversely, PE/2 does not inhibit PTP1B in vitro but increases the insulin effect in INSR and AKT phosphorylation in liver cells, thus acting as an insulin-sensitizing agent. These important differences between the two PE-derived compounds may be explained not only by dissimilarities in the underlying mechanisms through which these compounds affect the cells but also by the characteristics of the different cell lines and their respective insulin cascade and permeability to the molecules. However, considering PE/2’s inability to act through PTP1B, we may suppose that the improvement of hepatic insulin signaling induced by this compound could be mediated by inhibition of IL6 (Figure 6). Whether PE and PE/2 directly regulate IL6 expression or indirectly, through some downstream mediators, is not clear and could be the subject of further research.

## 4. Materials and Methods

### 4.1. Materials

Media, sera, and antibiotics for cell culture were purchased from Invitrogen (Grand Island, NY, USA). Protein electrophoresis and real-time PCR reagents were from Bio-Rad (Richmond, VA, USA). Western blotting and ECL reagents were from Amersham Biosciences (Arlington Heights, IL, USA). Sigma-Aldrich (Saint Louis, MO, USA) supplied solvents, deuterated solvents, and commercial reagents. All ^1^H NMR (700 MHz) analyses were carried out on a Bruker Avance Neo spectrometer (Bruker BioSpin Corporation, Billerica, MA, USA); chemical shifts were referenced to the residual solvent signal (CD_3_OD: *δ*_H_ = 3.31, *δ*_C_ = 49.0). The high-resolution MS spectra (negative mode) of PE, PE/2, and PE/3 were recorded by direct infusion into the ESI source of a Thermo LTQ Orbitrap XL mass spectrometer (Thermo-Fisher, San Josè, CA, USA). MeOH was used as the solvent for the infusion. HPLC analyses were performed on a Knauer Azura Pump 4.1 S instrument equipped with a Knauer K-2301 RI detector (LabService Analytica s.r.l., Anzola dell’Emilia, Italy).

### 4.2. Collection, Extraction, and Fraction of the Ascidian S. elegans

Exhaustive extraction and purification procedures of specimens of *S. elegans* collected in the bay of Pozzuoli (Naples, April 2021, 40°49′39.61″ N 14°09′11.56″ E) allowed the obtaining of PE in pure form. The identification of the organism was performed by Mr. Arturo Facente, and a voucher of this ascidian is deposited at the Department of Pharmacy, University of Naples Federico II, Napoli, Italy. The extraction was performed according to our already reported protocol [17,19] affording three organic extracts with different polarity grades (butanol, ethyl acetate, and water extracts). The butanol-soluble material (452.4 mg) underwent chromatographic separation by MPLC on reversed phase silica gel (C-18) using an increasing gradient elution: H_2_O/MeOH 9:1 → H_2_O/MeOH 7:3 → H_2_O/MeOH 1:1 → H_2_O/MeOH 3:7 → MeOH 100% → MeOH/CHCl_3_ 7:3 → MeOH/CHCl_3_ 1:1 → CHCl_3_ 100%. Our literature [17,18] and a preliminary ^1^H NMR analysis suggested that PE was the main constituent of the fraction eluted with H_2_O/MeOH 3:7 (*v*/*v*). Thus, this fraction was further chromatographed by RP-HPLC on a Synergy RP-MAX 4 μm column with H_2_O/MeOH 2:8 plus 0.1% of trifluoroacetic acid as the mobile phase, yielding PE (t_R_ = 20.2 min, 41.6 mg) in a pure state. The identity of PE was confirmed by comparing its NMR and HR-ESIMS data with our reported literature (Figures S1 and S2) [17,18]. HRESIMS *m*/*z* 668.4095 [M-H]^−^ (calcd. for C_32_H_63_NO_11_P 668.4139). 

### 4.3. Synthesis of PE/2 and PE/3 by Oxidative Cleavage of the Natural Metabolite

The fragments PE/2 and PE/3 were obtained by our semisynthetic approach on the marine derivative PE since the latter underwent the oxidative cleavage of the 1,2-diol system [17,22]. Indeed, a portion of PE (19.8 mg) was dissolved in 3.0 mL of a methanolic NaIO_4_ solution (0.01 M) and kept under magnetic stirring at room temperature (rt) for 3 h. After cooling the reaction mixture at 0 °C, a large excess of NaBH_4_ (12.0 mg) was added and stirred for an additional 1 h. Then, the reaction was warmed until rt, and partitioned (2 × 20 mL) between water and butanol. The collected organic phases were combined, dried over Na_2_SO_4_, filtered, and concentrated in vacuo. The crude material was purified by RP-HPLC (Luna C18 3 µm column, H_2_O/MeOH 2:8 + 0.1% TFA, flow rate 0.5 mL/min), yielding compounds PE/2 (6.3 mg, t_R_ = 2.3 min) and PE/3 (8.5 mg, t_R_ = 20.3 min) in their pure form. Their identity was unequivocally confirmed by comparing spectroscopic data with available literature (Figures S3–S6) [17,22]. HRESIMS for PE/2 *m*/*z* 274.1644 [M-H]^−^ (calcd. for C_13_H_24_NO_5_ 274.1649). HRESIMS for PE/3 *m*/*z* 395.2578 [M-H]^−^ (calcd. for C_19_H_40_O_6_P 395.2557).

### 4.4. Cell Culture Procedures and Cell Proliferation

HepG2 hepatocarcinoma cells were purchased from the American Type Culture Collection (ATCC, Manassas, VA, USA). HepG2 were cultured at 37 °C in a humidified 95% air and 5% CO_2_ atmosphere (all vol./vol.) and grown in Dulbecco’s modified Eagle’s medium (DMEM) supplemented with 10% fetal calf serum, 2% L-glutamine, 10,000 units/mL penicillin, and 10,000 μg/mL streptomycin. After overnight starvation, cells were incubated for 4 h with PE, PE/2, and PE/3 (25 µM) and then stimulated with insulin (100 nM) for the indicated times. 

### 4.5. Western Blot Analysis

Total cell lysates were obtained and separated by SDS-PAGE. Briefly, cells were solubilized with lysis buffer containing 50 mM HEPES, 150 mM NaCl, 10 mM EDTA, 10 mM Na_4_P_2_O_7_, 2 mM sodium orthovanadate, 50 mM NaF, 1 mM phenylmethylsulfonyl fluoride, 10 μg/mL aprotinin, 10 μg/mL leupeptin, pH 7.4, and 1% (*v*/*v*) Triton X-100. Lysates were clarified by centrifugation at 14,000 rpm for 20 min at 4 °C. The protein concentrations in the cell lysates were measured using a Bio-Rad DC (detergent-compatible) assay. Proteins (50 µg) were resolved by dodecyl sulfate-polyacrylamide gel electrophoresis (SDS-PAGE), transferred onto PVDF membrane, and blocked with 5% BSA in Tris-buffered saline containing 1% tween 20. Membranes were incubated with specific primary antibodies:

INSR (1:1000, Catalog #: 3025, Cell Signaling Technology, Beverly, MA, USA). pINSR (1:1000, Catalog #: 3021, Cell Signaling Technology, Beverly, MA, USA); AKT (1:1000, Catalog #: 06-558, Upstate Biotech, New York, NY, USA); pAKT (1:1000, Catalog #: 4058, Cell Signaling Technology, Beverly, MA, USA); ERK1/2 (1:1000, Catalog #: sc-514302, Santa Cruz Biotechnology, Dallas, TX, USA); pERK1/2 (1:1000, Catalog #: 9101, Cell Signaling Technology, Beverly, MA, USA); Vinculin (1:1500, Catalog #: sc-73614, Santa Cruz Biotechnology, Dallas, TX, USA). Secondary antibodies were all diluted 1:1000 and were anti-rabbit (Catalog #: 170-6515, Bio-Rad, Hercules, CA) and anti-mouse (Catalog #: 170-6516, Bio-Rad, Hercules, CA, USA).

### 4.6. Luminex Assay

HepG2 cells were seeded in serum-free media, and after 24h, conditioned media were collected and screened for the concentration of interleukin (IL)1β, IL1ra, IL6, IL8, IL6R, CCL2, CCL3, Inhibin, and VEGF, which were determined using a custom Human Magnetic Luminex Assay (R&D System) according to the manufacturer’s protocol. The magnetic bead-based assay was performed on a Luminex 200 System (Austin, TX, USA) with xPONENT Software 4.3.

### 4.7. Real-Time RT-PCR Analysis

Total cellular RNA was isolated from HepG2 cells by Qiazol reagent lysis (QIAGEN Sciences, Hilden, Germany), according to manufacturer instructions. Then, 1 µg of cell RNA was reversely transcribed using Superscript III Reverse Transcriptase (Life Technologies, Carlsbad, CA, USA). PCR reactions were analyzed using IQTM SYBR Green Supermix (Bio-Rad, Hercules, CA, USA). Reactions were performed using Platinum SYBR Green qPCR Super-UDG using an iCycler IQ Multicolor Real-Time PCR Detection System (Biorad, Hercules, CA, USA) running a total of 40 cycles. All reactions were performed in triplicate, and expression data were normalized to the geometric mean of the housekeeping gene beta-ACTIN and analyzed using the 2^−ΔCT^ or 2^−ΔΔCT^ methods [44]. The primer sequences used are described in electronic Supplementary Material Table S1. 

### 4.8. Determination of Glycogen Content

Glycogen was isolated from HepG2 cells homogenized in 0.1% SDS and saturated with Na_2_SO_4_ for 30′ at 37 °C, followed by EtOH precipitation. Glycogen content was determined as described in [45,46].

### 4.9. Statistical Procedures

Data were analyzed with GraphPad Prism 8.0 (GraphPad Inc., San Diego, CA, USA). Multiple comparisons among more groups were made using the ANOVA test with Tukey correction (for normally distributed data). *p* values equal to or less than 0.05 were considered statistically significant.

## 5. Conclusions

Overall, these results constitute an important contribution to the design of new ligands for various targets involved in the insulin signaling cascade and, consequently, to the search for new drug lead candidates. The current data also provides new insights into the capacity of the polyketide structures PE and PE/2 to improve hepatic insulin signaling and glucose homeostasis, suggesting IL6 as a possible pharmacological target. Although further investigation is required, our findings suggest that PE/2 could be a novel antidiabetic candidate with more relevant chemical accessibility than that of both PE and PE/3, even more so considering that it only possesses one stereogenic center. For that aim, the scaffold of PE/2 can be further manipulated towards the development of new agents for the treatment of hepatic metabolic diseases. 

## Figures and Tables

**Table 1:** Concentration (pg/mL) of cytokines, chemokines, and growth factors in culture medium obtained from HepG2 cells in the presence of PE and its derivates. Results are expressed as mean ± standard deviation. Asterisk denotes statistical differences (* *p* < 0.05).

	CTRL	PE	PE/2	PE/3
IL1β	1.02 ± 0.708	0.940 ± 0.589	1.23 ± 0.89	0.94 ± 0.59
IL1RA	16.0 ± 13.7	12.1 ± 6.78	11.0 ± 7.49	11.8 ± 11.5
IL6	6.55 ± 0.39	4.82 ± 0.40 *	4.90 ± 1.11 *	6.67 ± 0.34
IL8	76.7 ± 17.5	55.6 ± 5.64	48.7 ± 17.4 *	62.4 ± 1.43
IL6R	33.3 ± 22.7	34.1 ± 19.2	16.3 ± 2.64	35.0 ± 23.1
CCL2	444 ± 126	376 ± 15.7	374 ± 320	611 ± 46.0
CCL3	6.24 ± 1.81	7.28 ± 1.80	11.0 ± 7.49	9.36 ± 1.78
INHIBIN	71.3 ± 13.9	65.7 ± 25.8	73.3 ± 26.6	67.7 ± 15.5
VEGF	178 ± 81.4	188 ± 94.4	161 ± 81.7	188 ± 88.6

## Data Availability

The data that support the findings of this study are available upon reasonable request.

## Supplementary Materials

The following supporting information can be downloaded at: https://www.mdpi.com/article/10.3390/ijms25116039/s1.
