# Supplementary material for: Marine-Derived Phosphoeleganin and Its Semisynthetic Derivative Decrease IL6 Levels and Improve Insulin Signaling in Human Hepatocellular Carcinoma Cells
Source: Int J Mol Sci. 2024 May 30;25(11):6039. doi: 10.3390/ijms25116039 (PMC11173279; doi:10.3390/ijms25116039)
Supplement: Supplementary file 1 [file ijms-25-06039-s001.zip › ijms-2997295-supplementary/ijms-2997295-supplementary.pdf]

Marine-derived phosphoeleganin and its semisynthetic derivative decrease IL6 levels and improve insulin signaling in human hepatocellular carcinoma cells.

Ayewa L. Agognon[1,‡], Marcello Casertano[2,‡], Alessio Vito[2], Sonia Orso[1], Serena Cabaro[1], Federica Mormone[1], Cristina Morelli[1], Giuseppe Perruolo[1], Pietro Formisano[1], Marialuisa Menna[2,*],Concetta Imperatore[2,‡] and Francesco Oriente[1,3,*,‡]

Table of content

Figure S1. ^{1}H NMR spectrum (600 MHz) of PE in CD_3OD 1

Figure S2. HRESIMS spectrum of PE 1

Figure S3. ^{1}H NMR spectrum (600 MHz) of PE/2 in CD_3OD 2

Figure S4. HRESIMS spectrum of PE/2 2

Figure S5. ^{1}H NMR spectrum (600 MHz) of PE/3 in CD_3OD 3

Figure S6. HRESIMS spectrum of PE/3 3

Table S1. Primer sequences used in Real-time RT-PCR analysis 3

Description of Cell incubation with High glucose 4

Description Cell incubation with Palmitic Acid 4

Figure S1. ^{1}H NMR spectrum (600 MHz) of PE in CD_3OD

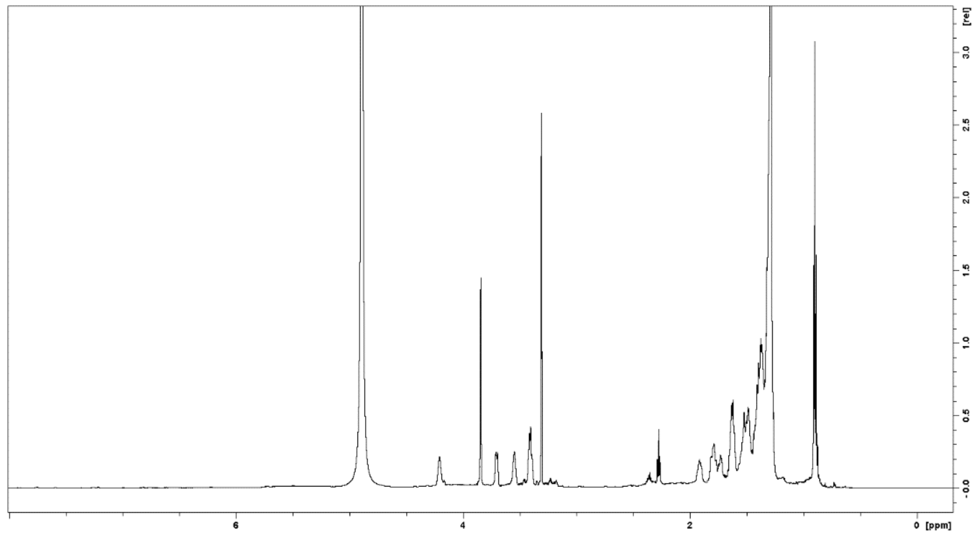

Figure S2. HRESIMS spectrum of PE

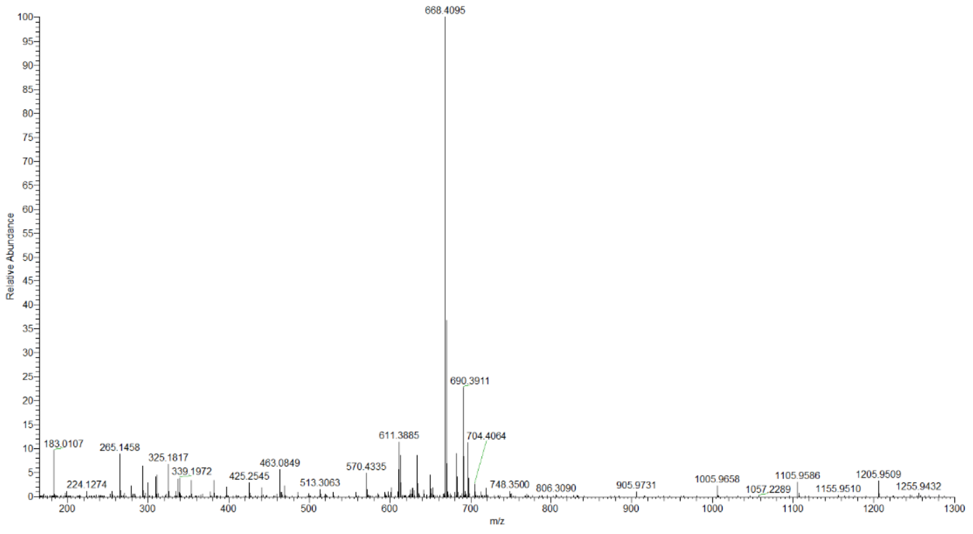

Figure S3. ^{1}H NMR spectrum (600 MHz) of PE/2 in CD_3OD

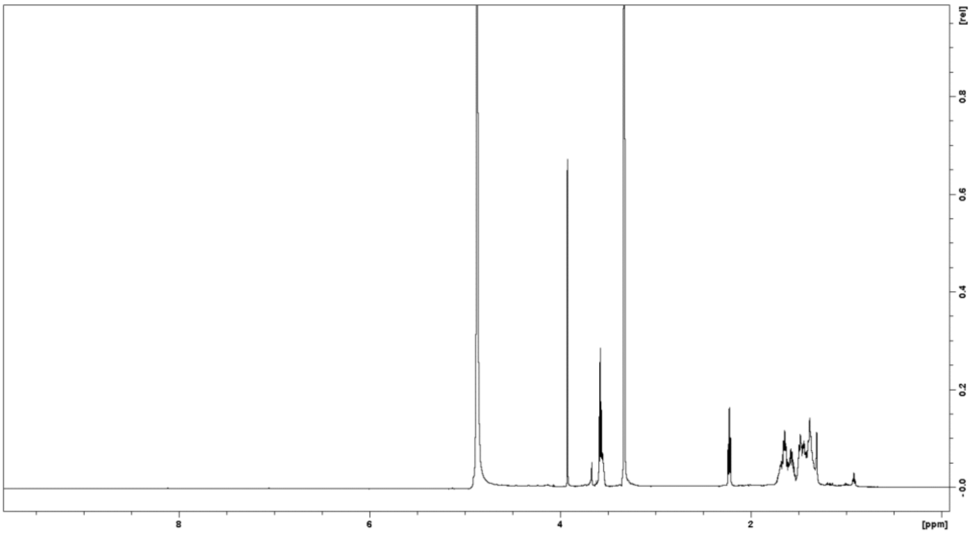

Figure S4. HRESIMS spectrum of PE/2

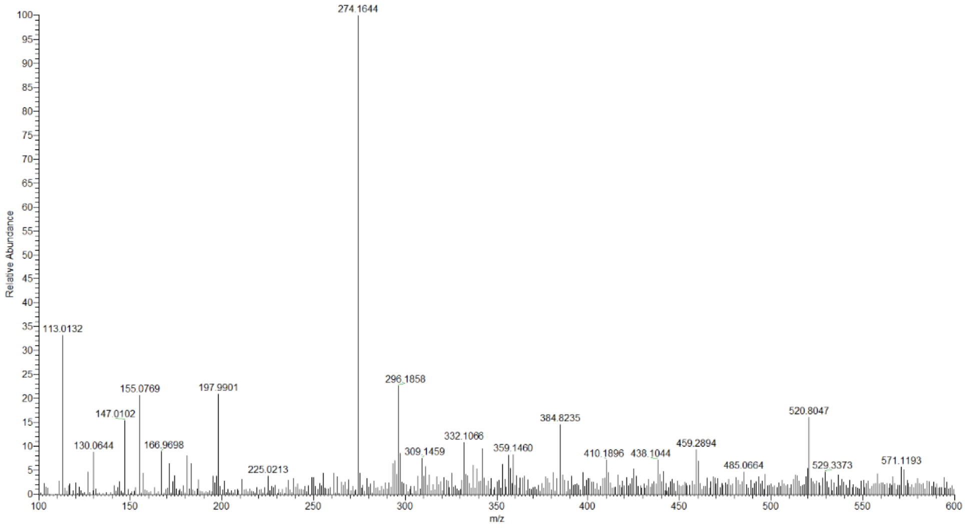

Figure S5. ^{1}H NMR spectrum (600 MHz) of PE/3 in CD_3OD

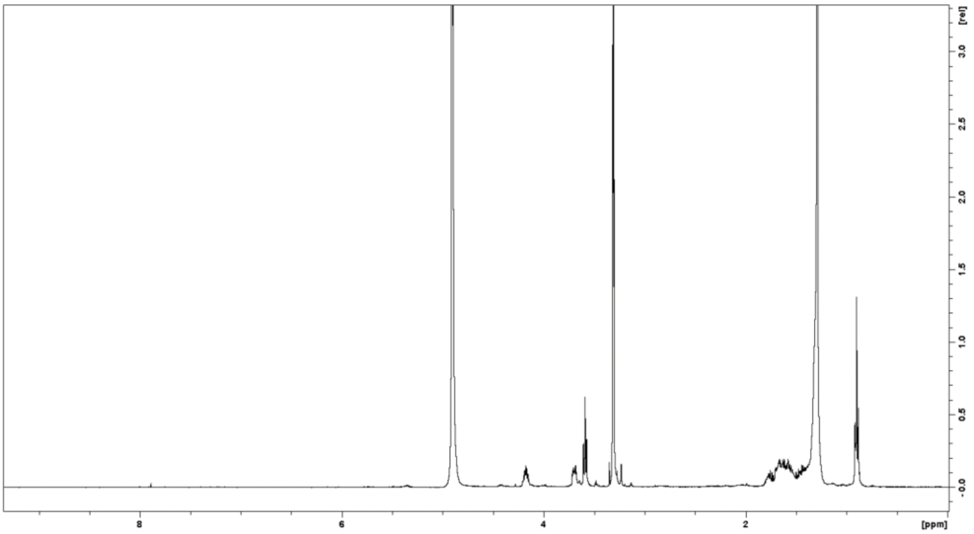

Figure S6. HRESIMS spectrum of PE/3

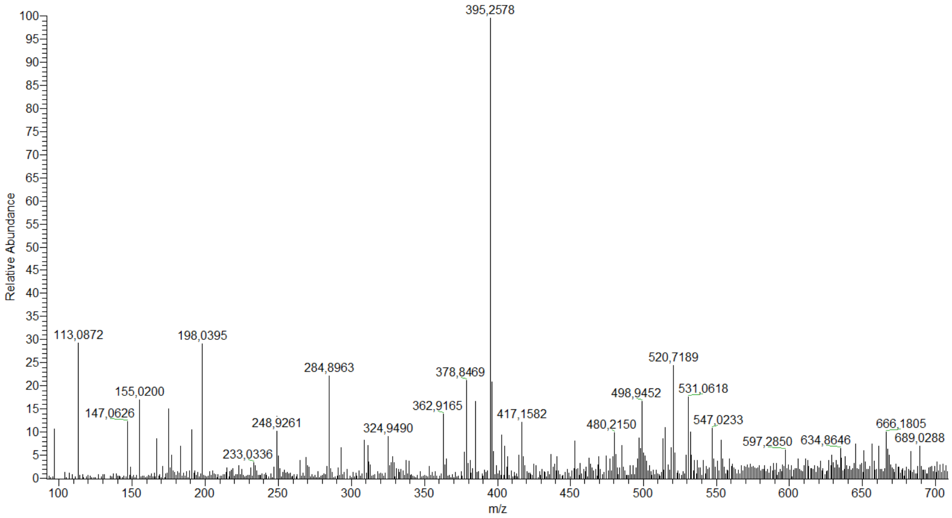

Table S1. Primer sequences used in Real-time RT-PCR analysis.

PRIMERS	SEQUENCES
PEPCK	Forward: 5'-CTGAACCTCTCGGCCAAAGT-3'<br>Reverse: 5'-TTTCGATCCTGGCCACATCC-3'
IL6	Forward: 5’-CAATGAGGAGACTTGCCTGGT-3’<br>Reverse: 5’-AGCTGCGCAGAATGAGATGA-3
β-ACTIN	Forward: 5'-GCGTGACATCAAAGAGAAG-3'<br>Reverse: 5'-ACTGTGTTGGCATAGAGG-3'

Cell incubation with High glucose

HepG2 cells were plated in 6 well plate (200000 cells/well) and grown as described in 4.4 for approximately 24h. Cells were then starved overnight with DMEM high glucose containing 0,25% BSA. The starvation medium was discarded, and fresh starvation medium enriched with glucose was added, to reach a final glucose concentration of 60mM and the compounds PE, PE/2, and PE/3 (25μM). After 24h of incubation, culture medium was collected for Luminex assay. Cells were washed with PBS/EDTA and recovered with trypsin. The pellet was washed with PBS 1% and resuspended in Qiazol for further RNA extraction.

Cell incubation with Palmitic Acid

To prepare PA stock, sodium palmitate was dissolved in NaOH 0,1M solution in a concentration of 25mM. The mixture was heated until complete solubilization at 70°C in a shaking water bath. The solution was then diluted with 10% FFA-free BSA-DMEM and heated at 55°C, to yield a final stock solution with 5mM PA. The final stock solution was filtrated before incubation with cells. HepG2 cells were plated in 6 well plate (200000 cells/well) and grown as described in 4.4 for approximately 24h. Cells were then starved overnight with DMEM high glucose containing 0,25% BSA. The starvation medium was then discarded, and fresh starvation medium was added to the cells with the addition of the PA stock, to reach a final concentration of 0,5mM and the compounds PE, PE/2, and PE/3 (25μM). Control cells treated only with NaOH in the same final concentration as working experiments.
